# *SNaPAfu*: A Novel Single Nucleotide Polymorphism Multiplex Assay for *Aspergillus fumigatus* Direct Detection, Identification and Genotyping in Clinical Specimens

**DOI:** 10.1371/journal.pone.0075968

**Published:** 2013-10-18

**Authors:** Rita Caramalho, Leonor Gusmão, Michaela Lackner, António Amorim, Ricardo Araujo

**Affiliations:** 1 Institute of Molecular Pathology and Immunology of the University of Porto, Porto, Portugal; 2 Faculty of Sciences, University of Porto, Porto, Portugal; 3 DNA Diagnostic Laboratory, State University of Rio de Janeiro, Rio de Janeiro, Brazil; 4 Division of Hygiene and Medical Microbiology, Innsbruck Medical University, Innsbruck, Austria; The University of Texas at San Antonio, United States of America

## Abstract

**Objective:**

Early diagnosis of invasive aspergillosis is essential for positive patient outcome. Likewise genotyping of fungal isolates is desirable for outbreak control in clinical setting. We designed a molecular assay that combines detection, identification, and genotyping of *Aspergillus fumigatus* in a single reaction.

**Methods:**

To this aim we combined 20 markers in a multiplex reaction and the results were seen following mini-sequencing readings. Pure culture extracts were firstly tested. Thereafter, *Aspergillus*-DNA samples obtained from clinical specimens of patients with possible, probable, or proven aspergillosis according to European Organization for the Research and Treatment of Cancer/Mycoses Study Group (EORTC/MSG) criteria.

**Results:**

A new set of designed primers allowed multilocus sequence typing (MLST) gene amplification in a single multiplex reaction. The newly proposed *SNaPAfu* assay had a specificity of 100%, a sensitivity of 89% and detection limit of 1 ITS copy/mL (∼0.5 fg genomic *Aspergillus*-DNA/mL). The marker A49_F was detected in 89% of clinical samples. The *SNaPAfu* assay was accurately performed on clinical specimens using only 1% of DNA extract (total volume 50 µL) from 1 mL of used bronchoalveolar lavage.

**Conclusions:**

The first highly sensitive and specific, time- and cost-economic multiplex assay was implemented that allows detection, identification, and genotyping of *A. fumigatus* strains in a single amplification followed by mini-sequencing reaction. The new test is suitable to clinical routine and will improve patient management.

## Introduction

*Aspergillus fumigatus* is the major filamentous fungal pathogen in severely ill patients, causing disseminated infections [1]. Invasive aspergillosis (IA) is associated with mortality above 50% [2]. Early and reliable diagnosis of invasive fungal infections (IFIs) can lower patients' mortality and reduce treatment expenses, due to an early targeted therapy, but this is often difficult and might be in part also due to the limitations of conventional diagnostic techniques (culture and histology), which often lack sensitivity, promptness, and efficiency [3]–[5]. As an example, detection of circulating galactomannan (GM) has become commonly used but false-positive and false-negative results are still a major limitation to this auxiliary method for an earlier IA diagnosis [6]. Nucleic acid-based assays have the capacity to diagnose fungal diseases in an early stage. False-positive results represent drawbacks, as a direct consequence of the high sensitivity of molecular assays and to ubiquitous presence of *A. fumigatus* in the environment [3], [7].

Molecular assays are presently recommended for correct identification of *A. fumigatus* within *Aspergillus* section *Fumigati* and evaluation of its genetic diversity, a critical issue for outbreak controlling [8]–[11]. Current gold standard in microbial genotyping is Multilocus sequence typing (MLST), developed for *A. fumigatus* by Bain et al. [12]. The present MLST panel combines seven housekeeping genes showing a discriminatory power of 0.93, not as high as microsatellite genotyping, but with the advantage of providing a free online database which improves data transferability and genetic analyses. MLST proved to be specific for this fungal species within section *Fumigati* [13]–[15], but remains expensive, laborious, and unfeasible for routine examination due to multiple sequencing.

The implementation of single nucleotide polymorphism (SNP) markers has been suggested as an attractive alternative for the detection of specific bacterial lineages and/or identification purposes [16]–[18]. A SNP based strategy has recently been developed for genotyping of *Pseudomonas aeruginosa* presenting a discriminatory power of 0.9993 comparing with MLST [19].

The aim of this study was to design an assay that combines detection, identification and genotyping in a single reaction and that performs directly on DNA extracts gained from clinical specimens. To this aim, we selected a panel of discriminatory-informative SNPs that are variable enough to accurately detect, identify and genotype *A. fumigatus* isolates and combine those markers in a single multiplex reaction named *SNaPAfu*.

## Materials and Methods

### Ethics Statement

The Ethics commission of Innsbruck Medical University had approved retrospective usage of residual materials from diagnostics. A written informed consent was provided by study participants and/or their legal guardians.

### MLST genotyping employing a multiplex strategy

The MLST sequence alignment generated by multiplex reaction is needed as basis for the development of the *SNaPAfu* assay, as this assay is built on SNPs located in MLST genes. A set of seven housekeeping genes was studied in 20 clinical and unrelated *A. fumigatus* isolates as suggested by Bain et al. [12]. These strains were obtained from sputum samples collected from patients admitted at Hospital S. João (Oporto, Portugal) and had been previously genotyped by microsatellite-based strategy [8], [9]. Singleplex amplification was performed as previously described [12]. These primers were found unsuitable for a multiplex reaction as they formed dimers. Therefore new primers were designed using Primer 3 (http://frodo.wi.mit.edu/) and the absence of hairpins and dimers verified in silico using Autodimer software (http://www.cstl.nist.gov/strbase/AutoDimerHomepage/AutoDimerProgramHomepage.htm); newly selected primers are listed in Table 1. Multiplex PCR amplification reaction consisted of: 0.5 µL of genomic DNA (50 ng–250 ng), 2.5 µL of My Taq™ HS MIX (Bioline) and 0.5 µL of primer mix (each primer at 2.0 µM), in a final volume of 5.0 µL. The multiplex PCR reaction was run at the following conditions: initial denaturation step at 95°C for 15 min, followed by 35 cycles of denaturation at 95°C for 30 s, annealing at 61°C for 1 min and extension at 70°C for 1 min, followed by a final extension step at 70°C for 10 min. Amplification products of multiplex PCR products were confirmed on polyacrylamide gel and visualized by silver staining.

**Table 1:** Primers used for MLST genotyping of *Aspergillus fumigatus* in a multiplex reaction.

Locus	Primer sequence (5′ to 3′)		Amplicon size (bp)
*ANXC4*	Forward	GCAAAGCGTGATGACAGAAA	582
	Reverse*	TAGGTCGACACAGGTTGTGG	
*BGT1*	Forward*	ACGGTGATGGCGTCAATAA	654
	Reverse	ACTGTCCCTCCTTCCGATCAA	
*CAT1*	Forward	TATATGACCGGCGAGCTCAA	822
	Reverse*	ACGAATCGGAAGGTATGCAC	
*LIP*	Forward	AGCGTAGCCTCCAGACATA	641
	Reverse*	CTCGCCTCACTTCTCCTCAG	
*MAT1-2*	Forward*	TCGACTTTCCAGAGCATGG	311
	Reverse	GTGGTCGCTTAATGACAGCA	
*SODB*	Forward	CAATGCACTCCTCGTCTCAA	826
	Reverse*	TTCTCCACAGCCTTCCAGTT	
*ZRF2*	Forward	AGGGACCTTGAGGATCGTC	698
	Reverse*	GATGATACCTTCGACCCATGA	

* Primer used for sequencing.

#### Sequencing analysis

Amplification products were purified with ExoSAP-IT (USB Corporation); 1.0 µL of ExoSAP-IT was added to 1.5 µL of amplification product. After purification, sequencing reaction was conducted as previously described [9]. Sequencing data were processed and analyzed with Sequencing 5.2 analysis software (Applied Biosystems).

### *SNaPAfu* assay

The methodic strategy used for the *SNaPAfu* assay is based on targeting only SNPs present in MLST genes instead of amplifying full MLST gene sequences. In the *SNaPAfu* assay these SNPs are amplified in a multiplex reaction and subsequently analyzed by mini-sequence reaction.

MLST sequences were added into a database that included all the information available at MLST database (http://pubmlst.org/afumigatus/). Sequence alignment of target genes was performed (throughout the 3036 bp MLST concatenated sequences) and a final set of 20 SNPs was selected. Primers were designed for those positions, combined in a multiplex reaction, and a non-homologous tail was added at the 5′ end of extension primers to ensure separation by capillary electrophoresis (Table 2).

**Table 2:** The primers used in *SNaPAfu* assay.

Primer#	Primer sequence	Tail added to the primer (bp)	Final primer size (bp)	Targeting base	Expected peak size (bp)§	Expected base*
A45_R	CGTATGAGAGTCCCTCGGAT	20	40	G/A	45–46	C/T
A49_F	CCTCGTCGTCCGTATCTGAGTAG	44	67	G/A	70–71	G/A
A313_R	ACCCGGACAGTTCCAGTATG	68	88	C/T	91	G/A
A392_F	GACGGGGGCACGAAGCC	0	17	G/A	19–20	G/A
B129_F	CAGCACCTTGCTCCCTCTC	16	35	G/A	40	G/A
B164_F	CGCTGCTGCCTCCAAGAA	6	24	T/C	35–36	T/C
C185_F	GGATTATCTGACCGGGTTCG	51	71	T/G	75	T/G
C193_R	GCATGGACCGCACGCTC	13	30	G/A	36–37	C/T
C403_R	ACCAACGTACCGAAATTGCC	65	85	C/G	87–90	G/C
C428_R	AAGACGATGTCAGTTGTGCG	28	48	G/A	53–54	C/T
C540_R	GGATCTCGTTGTCACCTCTGG	54	75	G/C	77	C/G
L54_R	CTGCCCCAATTAGCGAGG	0	18	C/G	31–32	G/C
L164_R	TTGGCTACTCGGCCTACTTC	35	55	G/A	58–59	C/T
L456_R	CTCCGATTCGAACCCAAATT	15	35	T/C	41	A/G
L487_R	GGACATCAATTCCGAGTTCGT	19	40	T/C	47	A/G
M15_R	GTATTGGCTGCTGGAGGTATG	40	61	G/A	63	C/T
S276_R	TTCCAAGCTGCCATATGTCTC	43	64	A/G	66	T/C
S329_F	GTAAGATGAACACCGCTTTGG	61	82	C/A	87	C/A
Z77_R	GCATCTTTCTCTCCATCCCAC	27	48	T/C	52–53	A/G
Z198_F	GCACCAGAAGAGTGTGAAGGC	34	55	C/T	58–59	C/T

# Primer nomenclature and incorporated information on the targeting polymorphic position (e.g. A45_R means that a reverse primer was designed in the polymorphic position 45 of the gene ANXC4 of MLST panel).

§ Expected peak size in the electropherogram (see Figure 2); bp means base pairs.

* Base expected in MLST and *SNaP* profiles; a complementary base is expected to be seen on the *SNaPAfu* electropherogram for primers designed for reverse sequence.

A genotyping reference set of 113 clinical and environmental *A. fumigatus* strains previously genotyped by microsatellite-based strategy isolated in Portugal were tested used for the establishment of the *SNaPAfu* assay [8], [9]. Clinical strains (n = 76) were mainly obtained from sputum samples collected from patients admitted at Hospital S. João (Oporto, Portugal). Environmental strains (n = 37) were collected from soil, water, and air samples at Oporto region. A reference strain of *A. fumigatus* (ATCC46646) was also included in the strains set. A second group of fungal strains belonging to the section *Fumigati* was obtained from Centraalbureau voor Schimmelcultures (CBS): *Aspergillus fumigatiaffinis* (CBS117186), *A. lentulus* (CBS116880, CBS117180, CBS117182, and CBS117885), *A. novofumigatus* (CBS117519), *A. unilateralis* (CBS126.56 and CBS283.66), *A. viridinutans* (CBS121595), *Neosartorya fischeri* (CBS316.89), *N. hiratsukae* (CBS124073), *N. pseudofischeri* (CBS208.92 and CBS110899), and *N. udagawae* (CBS114217). In addition, eight strains of *Aspergillus* belonging to other sections (*A. flavus*, *A. niger*, *A. nidulans*, and *A. terreus*) were also tested to verify the specificity of the assay. DNA was extracted from conidia using the sodium hydroxide method as previously described (http://www.aspergillus.org.uk/indexhome.htm?secure/laboratory_protocols). DNA extracts with concentrations reaching from 50 ng to 250 ng were suspended in 50 µL of ultrapure water (Qiagen) and stored at −20°C.

The mini-sequencing assays were carried out in a final volume of 5.0 µl, containing 1.5 µl of purified PCR product, 1.0 µl of the *SNaPAfu* primer mix (each primer at 1 µM), 1.0 µl of SNaPshot™ Ready Mix (Applied Biosystems), and 1.5 µl of ultrapure water (Qiagen). The mini-sequencing reaction was performed as recommended by the manufacturer. A post-purification treatment of SNaPshot™ products was performed with 1 U of SAP (USB Corporation) at 37°C for 1 h, followed by a cycle of 85°C for 15 min. The products (0.5 µl) were mixed with 9.0 µl of HiDi™ formamide (Applied Biosystems) and 0.5 µl of GeneScan-120 LIZ size standard (Applied Biosystems). Capillary electrophoresis was performed on a 3130xl Genetic Analyzer and the *SNaPAfu* profiles analyzed using GeneMapper™ version 4.0 (Applied Biosystems).

The detection limit of the newly proposed method for *A. fumigatus* in clinical samples was determined by testing serial dilutions of conidia (from 10000 to 0.01 *A. fumigatus* ITS copies/mL) in serum samples, as previously recommended [20]. By definition the lowest concentration of conidia capable to be detected by at least five molecular markers defined the detection limit. These tests were run in duplicates for two different strains.

### Application on clinical samples

A defined and well-described reference set of 37 clinical specimens (bronchoalveolar lavages (BAL) and biopsy materials) from Innsbruck Medical University were studied retrospectively using the new *SNaPAfu* assay (see Table 3). The samples were obtained from 21 patients with possible (n = 8), probable (n = 7), and proven (n = 6) aspergillosis according to the European Organization for the Research and Treatment of Cancer/Mycoses Study Group (EORTC/MSG) criteria [21]. The samples had been previously tested by culturing, direct microscopy, GM, and pan-fungal PCR. *Aspergillus* Platelia test (Bio-Rad Laboratories) was performed for BAL samples with sufficient sample volume according to manufactures instructions, regarding samples with values ≥0.5 as positive. An amount of 1 mL of BAL (total amount received for microbial diagnostics 50 mL) and 2.5 mg of lung biopsy was used for DNA extraction. BAL and biopsy samples were conserved with liquid nitrogen (−80°C) and stored until use. DNA was extracted using a modified cetyltrimethylammonium bromide protocol [13], [15], [22] and finally resuspended in 50 µl of ultrapure water. Extracted DNA was detected with an in-house pan-fungal PCR as previously described [22], [23]. Following DNA extraction, the DNA was stored at −20°C for few months (in this study the oldest samples were stored for 6 months).Sequencing was done with Big Dye terminators and a capillary sequencer (3500 Genetic Analyzer; Applied Biosystems).

**Table 3:** Clinical samples considered in this study and methods used for diagnosis of invasive aspergillosis (ARDS: Acute Respiratory Destress Syndrome; BAL: bronchoalveolar lavages; BS: skin biopsy samples; nm: not enough material to be tested; GM: galactomannan; PF-PCR: Pan-fungal PCF; (+) and (−) represent positive and negative results).

Patient	Gender	Age	EORTC	Disease	Hospital	Sample	Microscopy	Culture	GM	PF-PCR	MLST gel	*SNaPAfu*	A49_F	B129_F	L164_R	M15_R	All markers
1	M	58	Possible	Pneumonia	Hematology	Lung biopsy	NEG	NEG	nm	POS	(++)	POS	(+)	(+)	(−)	(+)	(−)
2	F	63	Possible	Pneumonia	ICU	BAL	NEG	Candida sp.	nm	POS	(+)	POS	(+)	(+)	(−)	(+)	(−)
3	F	70	Proven	Pneumonia	Pneumology	Lung biopsy	POS	*A. fumigatus*	nm	POS	(++)	POS	(+)	(+)	(+)	(+)	(+)
3						Lung biopsy	POS	*A. fumigatus*	nm	POS	(−)	POS	(+)	(+)	(−)	(+)	(−)
3						BAL	POS	*Mycelium sterilium*	nm	POS	(−)	POS	(+)	(−)	(−)	(+)	(−)
3						BAL	POS	*Mycelium sterilium*	nm	POS	(−)	POS	(+)	(+)	(−)	(+)	(−)
3						BAL	NEG	*Mycelium sterilium*	nm	POS	(++)	POS	(+)	(+)	(+)	(+)	(+)
3						BAL	NEG	*Mycelium sterilium*	nm	POS	(++)	POS	(+)	(+)	(+)	(+)	(+)
3						BAL	POS	NEG	nm	POS	(++)	POS	(+)	(+)	(+)	(+)	(+)
4	M	42	Proven	Pneumonia	Transplant	Abscess	POS	*A. fumigatus*	nm	POS	(+)	POS	(+)	(+)	(−)	(−)	(−)
4						Abscess	POS	*A. fumigatus*	nm	POS	(++)	POS	(+)	(+)	(+)	(+)	(−)
5	M	54	Possible	ABPA	Transplant	BAL	NEG	*A. niger*	POS	POS	(+)	POS	(+)	(+)	(−)	(+)	(−)
6	F	67	Possible	Pneumonia	ICU	BAL	NEG	NEG	NEG	POS	(++)	POS	(+)	(−)	(+)	(+)	(−)
7	F	62	Probable	Pneumonia	ICU	BAL	POS	*C.albicans, A. fumigatus*	POS	POS	(+)	POS	(+)	(+)	(+)	(−)	(−)
7						BAL	POS	Candida sp.	POS	POS	(−)	POS	(+)	(−)	(+)	(−)	(−)
8	M	65	Possible	Pneumonia	ICU	BAL	NEG	NEG	POS	POS	(++)	POS	(+)	(−)	(+)	(+)	(−)
8						BAL	NEG	NEG	NEG	POS	(++)	POS	(+)	(−)	(+)	(+)	(−)
8						BAL	NEG	NEG	nm	POS	(+)	POS	(+)	(−)	(−)	(−)	(−)
9	M	56	Possible	Pneumonia	Transplant	BAL	POS	*A. terreus*	POS	POS	(++)	POS	(+)	(−)	(+)	(+)	(−)
10	M	55	Proven	Pneumonia	Transplant	Lung biopsy	POS	NEG	POS	POS	(−)	POS	(+)	(−)	(+)	(+)	(−)
11	M	54	Proven	Pneumonia	Transplant	Lung biopsy	POS	*A. fumigatus*	nm	POS	(−)	POS	(+)	(+)	(+)	(+)	(−)
12	M	60	Probable	ARDS	ICU	BAL	POS	*Mycelium sterilium*	POS	POS	(++)	POS	(+)	(+)	(+)	(+)	(+)
13	F	63	Probable	Pulmonary infection	ICU	BAL	POS	*C. albicans, A. fumigatus*	POS	POS	(+)	POS	(+)	(+)	(+)	(+)	(−)
13					ICU	BAL	POS	*A. fumigatus*	POS	POS	(+)	POS	(+)	(−)	(−)	(+)	(−)
13					ICU	BAL	POS	*A. fumigatus*	POS	POS	(++)	POS	(+)	(+)	(+)	(+)	(+)
13					ICU	BAL	POS	*A. fumigatus*	POS	POS	(−)	POS	(+)	(−)	(+)	(+)	(−)
13					Medicine	BAL	POS	*A. fumigatus*	POS	POS	(−)	NEG	(−)	(−)	(−)	(−)	(−)
13					ICU	BAL	POS	NEG	POS	POS	(++)	POS	(+)	(−)	(+)	(+)	(−)
14	M	47	Probable	Pulmonary infection	Other	BS	POS	NEG	POS	POS	(++)	POS	(+)	(−)	(+)	(+)	(−)
15	M	52	Probable	Pneumonia	ICU	BAL	POS	NEG	POS	POS	(−)	NEG	(−)	(−)	(−)	(−)	(−)
16	M	68	Proven	Pneumonia	Pneumology	Lung biopsy	POS	NEG	nm	POS	(−)	POS	(+)	(−)	(+)	(+)	(−)
17	F	32	Possible	Pneumonia	ICU	BAL	NEG	*Candida sp.*	NEG	POS	(−)	POS	(+)	(−)	(+)	(−)	(−)
18	F	49	Probable	Pneumonia	Pneumology	BS	POS	*A. fumigatus, Candida* sp.	POS	POS	(−)	NEG	(−)	(−)	(−)	(−)	(−)
19	F	47	Possible	Pneumonia	Transplant	BAL	NEG	*A. fumigatus*	nm	POS	(−)	POS	(+)	(−)	(−)	(−)	(−)
19						BAL	POS	*C. albicans*	POS	POS	(−)	POS	(+)	(−)	(+)	(−)	(−)
20	M	69	Probable	Pneumonia	Medicine	BAL	POS	NEG	NEG	POS	(−)	POS	(+)	(+)	(+)	(+)	(−)
21	M	73	Proven	Aspergilloma	Medicine	Lung biopsy	POS	*A. fumigatus*	nm	POS	(−)	POS	(−)	(+)	(+)	(+)	(−)

The DNA extracts gained from the clinical samples were exposed to an enrichment pre-cycle with illutra™ GenomiPhi V2 DNA Amplification Kit (GE Healthcare) by following manufacturer's instructions before multiplex gene amplification. *SNaPAfu* assay were applied in these samples as described above.

### Data and statistical analysis

MLST sequencing data were deposited on an in-house record that included the information of 29 profiles attainable at online databases: 27 profiles accessible by MLST database (http://pubmlst.org/afumigatus/) and sequences of AF293 and AF1163 strains available at ENSEMBL (http://fungi.ensembl.org/index.html). The programs BioEdit sequence alignment editor (available at http://www.mbio.ncsu.edu/bioedit/bioedit.html) and Geneious™ Pro 5.3.4 (Biomatters Ltd, Auckland, New Zealand) were used to align and detect polymorphic positions in DNA sequences. Simpson's diversity index was applied to determine the discriminatory power of the proposed strategy. Data and statistical analyses were performed using Arlequin software v.3.0 (http://cmpg.unibe.ch/software/arlequin3/).

## Results

### Multiplex for amplification of MLST sequences

**Figure 1:**
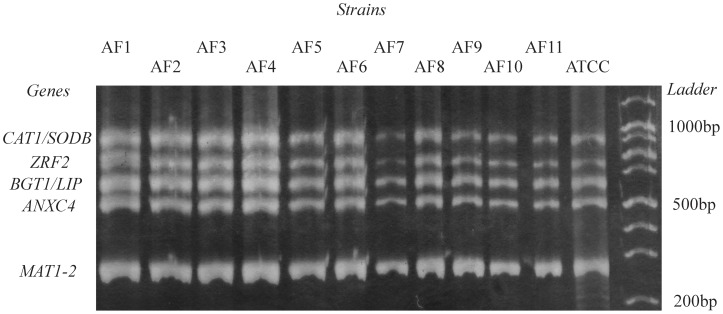
Silver stained polyacrilamide gel showing the band patterns of MLST multiplex amplification. (AF1 to AF11 are *A. fumigatus* strains from Portuguese collection; reference *A. fumigatus* ATCC 46645 was added).

The MLST sequences served us as basis for *SNaPAfu* assay, as the same gene fragments were targeted. To this aim we designed a new set of primers for MLST amplification in order to get MLST genes in a single multiplex reaction (see Figure 1). An optimal annealing temperature of 61°C was determined by performing gradient PCR (data not shown). The PCR multiplex co-amplified seven fragments, corresponding to the MLST housekeeping genes, with sizes ranging from 311 bp to 826 bp for all *A. fumigatus* strains.

### *SNaPAfu* assay

For this aim, sequences generated with the MLST assay were compared with online MLST data. We created an alignment for the seven genes. On the basis of this alignment we identified 61 polymorphic positions as possible targets for the new molecular assay. Non-polymorphic positions were eliminated, as well as redundant polymorphism positions, and a final set of 20 SNPs was selected. SNPs were selected for the *SNaPAfu* assay based on their differentiation power. Primers were tested independently in the *A. fumigatus* reference strain to confirm its specificity (a single peak was observed for each primer confirming the mini-sequencing reaction). Comparing the sequence types distinguishable by MLST versus *SNaP* profiles obtained by *SNaPAfu* assay, two MLST profiles (ST14 and ST27) could not be separated; markers for those profiles were not included in our panel due to primers hairpin formation.

*SNaPAfu* assay was tested on 113 clinical and environmental strains of *A. fumigatus*, as well as on the non-*Aspergillus fumigatus* set of clinical relevant fungi. *SNaPAfu* assay was successfully tested in all *A. fumigatus* strains, while in non-*fumigatus* strains failed to provide the complete set of 20 markers. Interestingly, a few markers could still be identified in strains belonging to section *Fumigati*: a) B129_F and M15_R in all strains from the section *Fumigati*, b) C193_R in *A. lentulus*, *A. viridinutans*, and *N. fischeri*, c) C540_R in *A. viridinutans*, *N. fischeri*, and *N. udagawae*, and d) C403_R and L164_R in *N. fischeri*. Strains outside section *Fumigati* did not show the presence of a single marker (Table S1).

**Figure 2:**
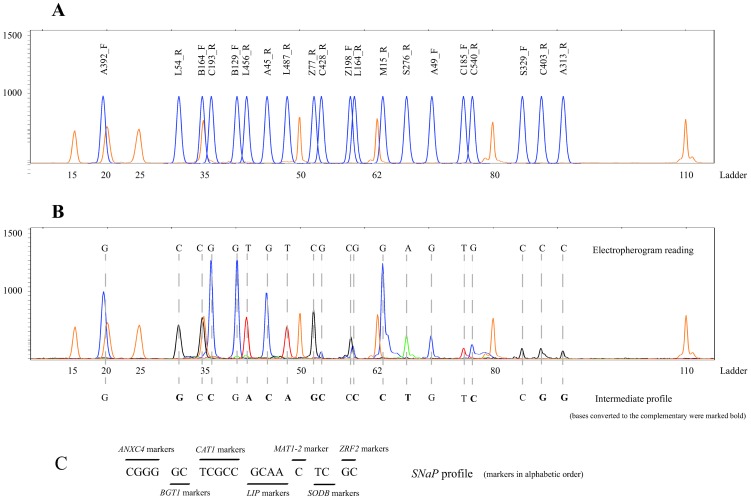
*SNaPAfu* assay: A) position of each marker on the automated electropherogram; and B) example of an *Aspergillus fumigatus* profile (peaks: orange – ladder; blue – guanine; black – cytosine; green – adenine; red – thymine; the interpretation of such the peaks gives the electropherogram reading, then converted to the intermediate profile where the results of the markers with the reverse primer were converted in the complementary base – markers marked bold were converted); and C) the *SNaP* profile of the isolate presented above obtained according to **Table 2** (e.g. A45_R, A49_F, … Z198_F) to facilitate comparison with MLST data – markers are presented in alphabetic order; when markers are amplified using reverse primers (e.g. A45_R) the complementary base should be included in the *SNaP* profile.

The final electropherogram for each *A. fumigatus* strain was converted into a *SNaP* profile (Figure 2). The *SNaP* profile represented the group of 20 polymorphisms obtained for each isolate (profiles were converted in accordance to MLST profiles to facilitate comparison of results, as shown in Figure 2). A set of 62 *SNaP* profiles was generated from our population of 113 strains; approximately 70% of the *SNaP* profiles were unique in the tested population of *A. fumigatus*. Few polymorphic positions included in our panel were exclusively described in the strain profiles available at MLST database (positions 129 of *BGT1* and 403 of *CAT1*), while others were uniquely described in our strains set (positions 392 of *ANCX4*, 185 and 540 of *CAT1*, 164 and 456 of *LIP*, and 329 of *SODB*). Strains with similar *SNaP* profile were re-sequenced by MLST scheme in order to assess the location and number of polymorphisms that could not be detected by *SNaPAfu* assay. A few polymorphisms were found in a small group of strains, mainly in the genes *ANCX4* and *CAT1*. Novel polymorphisms were uncommon and detected in strains easily differentiated by microsatellites. Those polymorphisms were not included later in the *SNaPAfu* assay, as they did not add new major discriminatory information.

For this newly developed method, a diversity index value of 0.99 was achieved when compared to MLST. These strains have been previously genotyped by a single microsatellite-based multiplex PCR [8], [9]; furthermore, D was recalculated for the arrangement of both genotyping methods (*SNaPAfu* plus microsatellite multiplex) offering a combined discriminatory power of 0.9998.

### Application of *SNaPAfu* on clinical samples

The detection limit of *SNaPAfu* assay was tested for two clinical strains. Serial dilutions of conidia were conducted as previously described [20] using ultrapure water (details in Table S2). The set of 20 markers was observed to be constantly obtained in samples with at least 10 *A. fumigatus* ITS copies/mL. Bellow this limit few markers failed. The value of 1 ITS copies/mL defined the detection limit of *SNaPAfu* assay as more than 5 markers can still be detected.

Clinical samples from 37 patients with possible/probable/proven according to the *Aspergillus* infection EORTC/MSG criteria were tested with *SNaPAfu* assay. Marker A49_F, confirmed above to be specific for *A. fumigatus*, was observed in 33 of 37 clinical samples (89%) confirming once again its ability to detect the present of this species (Table 3). Complete *SNaPAfu* profiles were observed in six samples (total DNA content ranging from 5 ng/µL to 375 ng/µL; among those were one lung biopsy and five BAL samples) retrieved from patients suffering from probable or proven aspergillosis according to the EORTC/MSG criteria (see Table 3). The positivity of direct microscopy and the other diagnostic tests already indicated the high fungal burden present in some samples, reinforced by the presence of highly marked bands on MLST electrophoresis. The results of direct microscopy, culture and GM showed agreement values of 62%, 46%, and 67%, respectively, with *SNaPAfu* results, similarly to pan-fungal PCR. Samples with efficient amplification of MLST genes and showing intense bands in MLST electrophoresis resulted in clearer electropherograms and better correlation with *SNaPAfu* results (Table 3). The samples with lower fungal DNA amount showed less intense bands or absence of bands in MLST electrophoresis and, therefore, only a few peaks (from 2 to 6) were observed on the final electropherogram. Markers B129_F, L_164R, and M15_R were commonly observed in clinical samples (were detected in around 70% of the samples), however, these markers were identified above in species from section *Fumigati* besides *A. fumigatus*.

## Discussion

The new *SNaPAfu* assay, an innovative method proficient to detect, identify and genotype *A. fumigatus* was established. This strategy of this assay is to target the most relevant polymorphisms present in MLST alignments. Instead of sequencing gene sequences like MLST, in the new *SNaPAfu* assay only SNPs are targeted, amplified in a multiplex reaction and subsequent analyzed by mini-sequence reaction. MLST has major advantages as described earlier [7]; disadvantages are undoubtedly related with the final cost (over €50 per sample), and the extended time needed to apply the method to large collections of strains. *SNaPAfu* assay offers informative data in a single amplification and mini-sequencing reaction. The *SNaPAfu* assay requires less than a working day (<8 h) to obtain results from clinical samples and estimated cost per sample is less than € 10 (depending on supplier). This innovative method can detect the presence of single or multiple *A. fumigatus* strains in the DNA extracts of clinical specimens, a pioneering aspect regarding molecular tools with diagnostic purposes, especially in clinical samples' studies such as those related to IA. Although *ITS* is currently recognized as the primary barcoding marker [24], it presents some limitations for identification of closely related species [25]. MLST genes are capable of reliable identification of several microorganisms and accumulate sufficient variability to genotype microbial isolates [13], [26]–[28]

The ability of *SNaPAfu* assay to detect the presence of *A. fumigatus* was confirmed on clinical samples. The specific molecular marker A49_F showed a great sensitivity (89%) in clinical samples. Some other markers included in the assay showed less sensibility (60–70%) to detect *A. fumigatus*, but the presence of several markers in the multiplex platform guarantees the presence of some peaks in the final electropherogram, even if a very small amount of DNA is present in the extract gained from bronchoalveolar or biopsy materials. *SNaPAfu* assay showed high sensitivity (89%) and good correlation with GM values (67%), similarly to previously described for other molecular assays [29], [30].

The presence of a non-*fumigatus Aspergillus* species that belongs to section *Fumigati* can also be suggested by the present methodology. Six markers (B129_F, C193_R, C403_R, C540_R, L_164R, and M15_R) were detected in species from section *Fumigati*, while the remaining markers were specific for *A. fumigatus*. The presence of such markers and the absence of specific *A. fumigatus* markers may suggest the presence of a rare species belonging to section *Fumigati*, some of them clinically very relevant and even resistant to the commonly used antifungal compounds [12], [13], [15], [31]. However, this ability to detect and confirm the presence of other species from section *Fumigati* still needs to be clarified. These species are rare and its genetic diversity is still poorly known. A higher number of species and strains need to be tested to confirm the persistent presence/absence of those molecular markers in their genome. No markers could be detected in other *Aspergillus* species.

The availability of molecular multiplexes for detection of several fungi has been reported of greater clinical interest, and it might be of major importance regarding molecular diagnosis approaches. An ideal molecular panel would be converted into an assay applicable to clinically complex samples and comprising a pool of markers suitable not only for detection, but also for identification and general overview of microbial population structure (if possible available through free online platforms). The novel *SNaPAfu* may be used as a feasible, reliable and forceful alternative regarding this matter.

Major limitations of the present assay are related to the difficulties to detect some markers in clinical samples. A complete panel with 20 peaks was observed only in samples with high DNA quality and quantity. As it has already been reported [32], the efficiency of the *Aspergillus* PCR is limited by the extraction procedure, such as low recovery of fungal DNA complemented with bacterial and human DNAs and/or presence of PCR inhibitors, and not by PCR amplification itself. The usage of molecular assays in such samples results in the loss of some genotyping power and the ability to detect multiple strains. Further inclusion or replacement of specific and well-studied markers may improve this feature. As previously stated, our method had a detection limit of 0.5 fg genomic *Aspergillus* DNA/mL, which is similar to 1 ITS copy/mL. This assay was not tested in blood and/or serum samples. Some limitations can be faced in such samples as the presence of PCR inhibitors and the DNA concentration can be below the detection limit observed in this study. Biopsies and BAL samples used in this work are currently very important clinical samples to establish proven invasive infections [21]. Improvements that could lead to the application of this tool in such samples would be of great value to clinical laboratories.

One of the major benefits of this assay is the possibility to reach fungal diagnosis, *A. fumigatus* identification and genotype in a single reaction. A clearer and deeper understanding of the *A. fumigatus* epidemiology and routes of transmission is essential to develop coherent and effective control measures to prevent contact of critical patients. Employment of reliable genotyping methods becomes crucial for understanding the large genetic diversity of *A. fumigatus* [8]–[10], [23], [33], monitoring clinical outbreaks caused by *A. fumigatus* [34], [35] and identifying contamination sources in hospitals. MLST is the current “gold standard” method for microorganism genotyping; *SNaPAfu* assay herein described exhibits similar discrimination power with great improvements in practicability and cost. The inherent plasticity of the current assay allows the addition of these and other markers to the panel able to provide relevant information concerning the studied fungal strains (e.g. pathogenicity, susceptibility, mating-type, or population genetics of specific populations). Such information coded by SNPs can easily be managed and included in the present panel.

Molecular methods are efficient to detect multiple strains in daily clinical samples and assess the efficacy of antifungal treatments [9], [36]–[38]. We strongly believe that *SNaPAfu* comprises a great potential for clinical routine examination launching a new generation of diagnostic molecular tools. It represents a fast, standardized, inexpensive and interactive platform for detection, identification and population analysis of *A. fumigatus* in clinical specimens.

## Supporting Information

Table S1 **Results of** ***SNaPAfu*** **assay tested in** ***Aspergillus*** **species.**
(XLSX)

Table S2 **The detection limit of** ***SNaPAfu*** **assay determined by testing serial dilutions of** ***A. fumigatus*** **conidia.**
(XLSX)
